# Genetic analysis of *Aedes aegypti* captured at two international airports serving to the Greater Tokyo Area during 2012–2015

**DOI:** 10.1371/journal.pone.0232192

**Published:** 2020-04-28

**Authors:** Kentaro Itokawa, Jinping Hu, Nayu Sukehiro, Yoshio Tsuda, Osamu Komagata, Shinji Kasai, Takashi Tomita, Noboru Minakawa, Kyoko Sawabe

**Affiliations:** 1 Pathogen Genomics Center, National Institute of Infectious Diseases, Tokyo, Japan; 2 Department of Vector Ecology and Environment, Institute of Tropical Medicine, Nagasaki University, Nagasaki, Japan; 3 Fukuoka Quarantine station, Fukuoka, Japan; 4 Department of Medical Entomology, National Institute of Infectious Diseases, Tokyo, Japan; 5 Antimicrobial Resistance Research Center, National Institute of Infectious Diseases, Tokyo, Japan; Fundacao Oswaldo Cruz Instituto Rene Rachou, BRAZIL

## Abstract

The introduction of exotic disease vectors into a new habitat can drastically change the local epidemiological situation. During 2012–2015, larvae and an adult of the yellow-fever mosquito, *Aedes aegypti*, were captured alive at two international airports serving the Greater Tokyo Area, Japan. Because this species does not naturally distribute in this country, those mosquitoes were considered to be introduced from overseas *via* air-transportation. To infer the places of origin of those mosquitoes, we genotyped the 12 microsatellite loci for which the most comprehensive population genetic reference is currently available. Although clustering by Bayesian and multivariate methods both suggested that all those mosquitoes captured at the airports in Japan belonged to the Asia/Pacific populations, they were not clustered into a single cluster. Moreover, there was variation in mitochondrial cytochrome oxidase I gene (*CoxI*) haplotypes among mosquitoes collected in different incidents of discovery which indicated the existence of multiple maternal origins. We conclude there is little evidence to support the overwintering of *Ae*. *aegypti* at the airports; nevertheless, special attention is still needed to prevent the invasion of this prominent arbovirus vector.

## Introduction

*Aedes aegypti*, dengue-yellow fever mosquito, distributes in the most part of the tropical and subtropical regions worldwide. This species has strong biting preference for humans and is adapted to urbanized environments, which render them an extremely effective vector for numerous arthropod-borne viral diseases including dengue, yellow fever and Zika fever. The current global distribution of this species is considered a result of the intercontinental movement of the mosquitoes along with trades and traffic by humans. Recent population genetics studies have indicated the existence of two major genetic clusters of *Ae*. *aegypti* worldwide [1,2]. The African cluster, which is distributed exclusively in Africa, is believed to represent the ancestral population of this species. Conversely, the *Ae*. *aegypti* population that is distributed in all regions outside of Africa, as well as some parts of Africa, is a monophyletic population lineage. Mosquitoes in this “out-of-Africa” cluster are more domesticated and well adapted to human inhabitation than are mosquitoes in the “Africa” cluster. The out-of-Africa cluster may have been transported from Africa to other parts of the world probably around the 16^th^ century along with transatlantic traffic [3].

**Fig 1:**
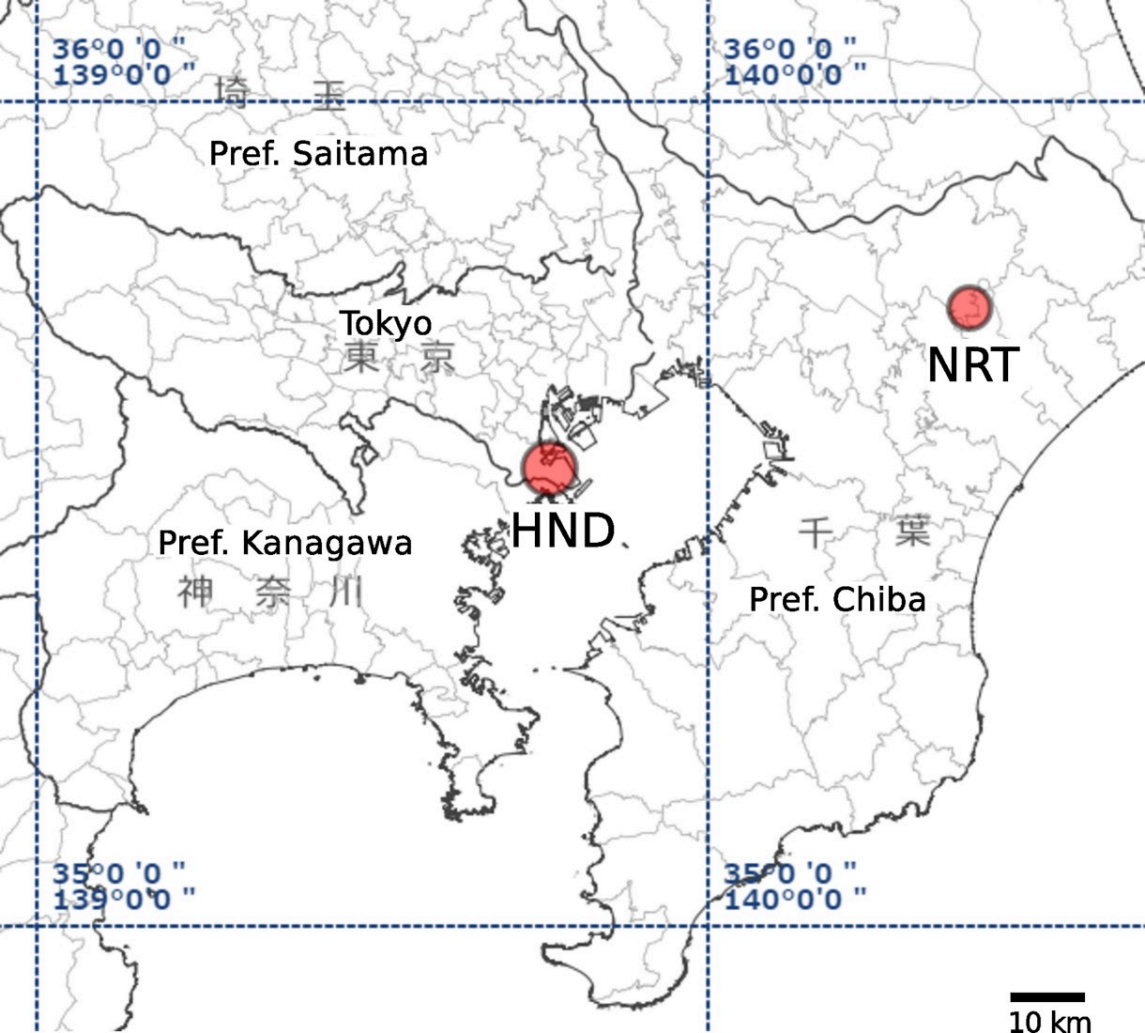
Locations of the Narita (NRT) and Haneda (HND) airports on a map including Tokyo and its peripheral cities. The map was reproduced from the Geospatial Information Authority of Japan website (https://www.gsi.go.jp). This map is licensed under the Government of Japan Standard Terms of Use (Ver.2.0). The Terms of Use are compatible with the Creative Commons Attribution License 4.0 (CC By).

Modern global transportation may accelerate the spread of such important insect pests across continents. Aircrafts is one of the most important pathway of spread of diseases for its daily volume and speed [4,5]. In Japan, the number of international scheduled flights has been increasing constantly in recent years (Japan Ministry of Land, Infrastructure, Transport and Tourism https://www.mlit.go.jp/koku/koku_fr19_000005.html), partly because of the rapid growth of low-cost carrier business. Thus, the reinforcement of surveillance systems for exotic mosquitoes within airports is highly demanded. In 2012, *Ae*. *aegypti* larvae were discovered in a single oviposition trap (ovitrap) placed in a passenger terminal of Narita International Airport (NRT), Chiba, Japan, which was the first detection of this mosquito species in a building of an international airport, in this country [6]. Insecticide was sprayed around the area soon after the discovery, and the follow-up intensive survey did not detect any additional *Ae*. *aegypti* during that season [6]. After this 2012 incident, however, the mosquitoes were sporadically captured again by ovitraps installed at NRT in August and September 2013, September 2014 and June, September and November 2015 (Table 1). Moreover, in September 2013, a single *Ae*. *aegypti* adult was captured at another airport, Tokyo International Airport (i.e. Haneda Airport: HND), which is located approximately 60 km south-west away of NRT (Fig 1). Although the continuous discoveries of *Ae*. *aegypti* in airports would represent repeated introductions from one or several foreign regions, we also considered the possibility of the existence of a source population of *Ae*. *aegypti* overwintering in the airport buildings, especially in the cases of NRT, where multiple incidents of discovery were recorded.

**Table 1:** Description of the incidents of capture of *Ae*. *aegypti* at the two international airports in Japan, during 2012―2015.

ID	Location	Period	Description*	N	*Cox1* Access. No.
NRT12	NRT	2012 Aug	Pupae and larvae were discovered in single ovitrap. Intensive survey after the discovery in area 400 m around did not detect additional mosquito (Sukehiro et al., 2013).	4	LC482631
HND13	HND	2013 Sep	Single adult male was captured in CDC light trap placed in cargo terminal. Intensive survey after the discovery in area 400 m around did not detect additional mosquito	1	LC482636
NRT13_Aug	NRT	2013 Aug	Pupae and larvae were discovered in single ovitrap set in airplane arrival terminal which located 1.5 km depart from NRT13_Sep_Aug discovered spot. Intensive survey after the discovery in 400 m area around the spot did not detect additional mosquito.	8	LC482632
NRT13_Sep	NRT	2013 Sep	Pupae and larvae were discovered in single ovitrap set in cargo terminal which located 1.5 km depart from NRT13_Sep_Aug discovered spot. Intensive survey after the discovery in 400 m area around the spot did not detect additional mosquito.	10	LC482633
NRT14	NRT	2014 Sep	Larvae were discovered in single ovitrap. Intensive survey after the discovery in 400 m area around the spot did not detect additional mosquito.	7	LC482630
NRT15_Jun	NRT	2014 Jun	Larvae were discovered in single ovitrap. Intensive survey after the discovery in 400 m area around the spot did not detect additional mosquito.	4	LC482634
NRT15_Sep	NRT	2014 Sep	Larvae were discovered in single ovitrap. Intensive survey after the discovery in 400 m area around the spot did not detect additional mosquito.	2	LC482629
NRT15_Nov	NRT	2014 Nov	Larvae were discovered in single ovitrap. Intensive survey after the discovery in 400 m area around the spot did not detect additional mosquito.	4	LC482635

*From Vector Surveillance Reports by the Quarantine Information Office, Ministry of Health, Labor and Welfare Japan: https://www.forth.go.jp/ihr/fragment2/index.html)

Preceding studies using genotype data for 12 microsatellite loci have revealed the hierarchical structure of the worldwide *Ae*. *aegypti* population [2,7]. According to the results of those studies, the worldwide *Ae*. *aegypti* population is divided into Africa and out-of-Africa clusters, as mentioned above. The out-of-Africa cluster is further divided into two New-World clusters and one Asia/Pacific cluster [7]. We considered that such a hierarchical structure and the existing comprehensive microsatellite genotype frequency data for this species [7] would allow us to narrow down the origin of the *Ae*. *aegypti* discovered in airports. In this study, we analyzed the genotypes of the 12 microsatellite loci and the mitochondrial cytochrome oxidase 1 (*Cox1*) gene haplotypes in the mosquito samples captured in airport buildings in the Tokyo area during 2012–2015.

## Material and methods

### Mosquitoes

Routine mosquito surveillances perfomed during 2012–2015 by the airport quarantine station stuff discovered *Ae*. *aegypti* larvae and adults in two international airports serving the Greater Tokyo Area (Fig 1) (Vector Surveillance Reports by the Quarantine Information Office, Ministry of Health, Labor and Welfare Japan: https://www.forth.go.jp/ihr/fragment2/index.html). Among the incidents listed in Table 1, the incident that occurred at NRT in 2012 has been described in detail in Sukehiro et al. (2013). Those mosquitoes were identified as *Ae*. *aegypti* morphologically. Some of the larvae were kept in the laboratory and grown to adults before being provided to us. For some mosquitoes, we obtained only one or a few legs from the quarantine office after the remider of their bodies was subjected to test for known flavivirus and Chikungunya virus by RT–PCR assay.

### DNA extraction

The modified alkaline lysis method [8] was used to prepare a PCR template from one to three legs of a single adult mosquito. In our modified alkaline lysis method, legs were homogenized in 10 μl of NaOH solution (0.2 M) in each well of 8-stripped PCR tubes by shaking with a zirconia bead (2 mm in diameter, Nikkato, Japan) in TissueLyser II (Qiagen) for 30 s at 30 Hz. The homogenate was incubated for 10 min at 75°C in thermal cycler, then neutralized by adding 10 μl of neutralization buffer (360 mM tris-HCl, 10 mM EDTA, pH 8.0) and 90 μl of Milli-Q water. This crude extract was used for subsequent PCRs for microsatellite and mitochondrial DNA.

### Genotyping of microsatellite loci

The 12 microsatellite loci developed by Brown et al. [2] and Slotman et al. [9] were amplified by PCR with labeled M13 primers as described in Brown et al. [2]. The primers and fluorescent dye combinations used here are described in S1 File. The PCR mixture contained 1 μl of template DNA, 1× Type-it Multiplex PCR Master Mix (Qiagen), 0.2 μM of each locus specific reverse primer, 0.02 μM of each locus specific forward primer and 0.2 μM of fluorescent-labeled M13 primer. The PCR conditions were 95°C for 2 min, 40 cycles of 98°C for 5 s, 55°C for 90 s and 72°C for 20 s, and a final extension on 72°C for 1 min. The resulting PCR fragments were electrophoresed with GeneScan 500 LIZ size standard (Applied Biosystems, ABI) on an ABI3130 instrument (ABI) for fragment analysis. Allele sizes were scored using the Peak Scanner Software v1.0 (Thermo Fisher Scientific). Five DNA samples previously analyzed by Brown et al. [2] were also genotyped in same manner to calibrate the consistency of allele calling between the different laboratories.

### Population clustering and assignment

The genotype data of airport populations were merged with the reference individual genotype table (VBP0000138 in Population Biology Project of VectorBase.org) (excluding the *Ae*. *mascarensis* and *Ae*. *queenslandensis* data) and formatted for analysis by STRUCTURE 2.3.4 [10,11]. Each run was conducted with 200,000 burn-in followed by 500,000 sampling, without using prior information of collection locations and using an allele frequency correlated model for 10 independent runs as replication. The best K value was determined according to Evanno’s criteria [12]. The replications at the best K were averaged by CLUMPP [13], and visualized by DISTRUCT [14] using the CLUMPAK server [15].

The discrimination analysis of principle component (DAPC) was conducted for microsatellite genotype data using the adegenet v2.0.1 package [16] in R v3.3.2. Countries of origins were used for the predefinition of populations for reference genotype panels. Hawaii and the other parts of US were treated as separated regions. For airport samples collected in Japan, samples collected in each different incident were treated as distinct populations.

Population assignment analyses were conducted in GeneClass2 [17]. First, the reference genotypes were divided into Africa and out-of-Africa groups. In self-assignment test, as setting cutoff threshold probability to 0.8, GeneClass2 assigned 95.4% African genotypes (42/918) and 98.0% out-of-Africa genotypes (2661/2714) back to each original group. The misassignment (assigning to wrong cluster with probability >0.8) rates, on the other hand, were 2.9 and 1% for African and out-of-Africa genotypes, respectively. Subsequently, the out-of-Africa reference genotypes were divided into New-World and Asia/Pacific groups. In the self-assignment test perfumed among these groups, GeneClass2 properly assigned 88.2% (1740/1972) New-world genotypes and 88.1% (654/742) Asia/Pacific genotypes back to each original group. Misassignments, on the other hand, occurred in 8.3% and 7.3% for New-World and Asia/Pacific genotypes, respectively.

### Sequencing of the cytochrome oxidase I (*Cox1*) gene in mitochondrial DNA

Fragments of the *Cox1* gene were amplified individually using primers COI-FOR (5ʹ–GTAATTGTAACAGCTCATGCA–3’) and COI-REV (5ʹ–AATGATCATAGAAGGGCTGGAC3’) [18]. The 10 μl reaction mixes contained 1 reaction buffer (Qiagen), 0.2 mM dNTP in final concentration, and 20 pmol of each primer, 1 U of Taq polymerase (Qiagen, USA) and 1 μl of the DNA template. PCR was performed under the following conditions: 94°C for 3 min, 35 cycles of 94°C for 15 s, 55°C for 30 s, and 72°C for 30 s; and final extension at 72°C for 10 min. The amplified PCR products were cleaned using ExoSAP-IT (USB Corporation, Cleveland, OH, USA) and sequenced in a 3730 DNA Analyzer (Applied Biosystems) using a BigDye Terminator v 1.1 Cycle Sequencing Kit (Applied Biosystems). The *Cox1* haplotype of NRT13_Sep was queried in a BLASTN [19] search at the NCBI Nucleotide collection (nt/rt) database (2019/05/09) restricted in *Ae*. *aegypti*, and hits with more than 95% query coverage were aligned using MUSCLE [20]. Sequences retrieved were AF380835, AF390098 and AY056597 [21]; AF425846 [22]; AY432106 and AY432648 [23]; EU352212; HQ688292-688298 [24], JQ926676-926684, JQ926686-926690, JQ926692-926696, JQ926698-926700 and JQ926702, JQ926704 [25]; KF909122 [26], KM203140-203248 [27]; KT313642, KT313645, KT313648, KT313650-313653 [28]; KT339661 and KT339679-339683 [29], KU186990; KX171382-171394. A haplotype network was drawn for the alignment using the *pegas* package (v0.11) in R [30].

## Results

### Population genetic analysis

**Fig 2:**
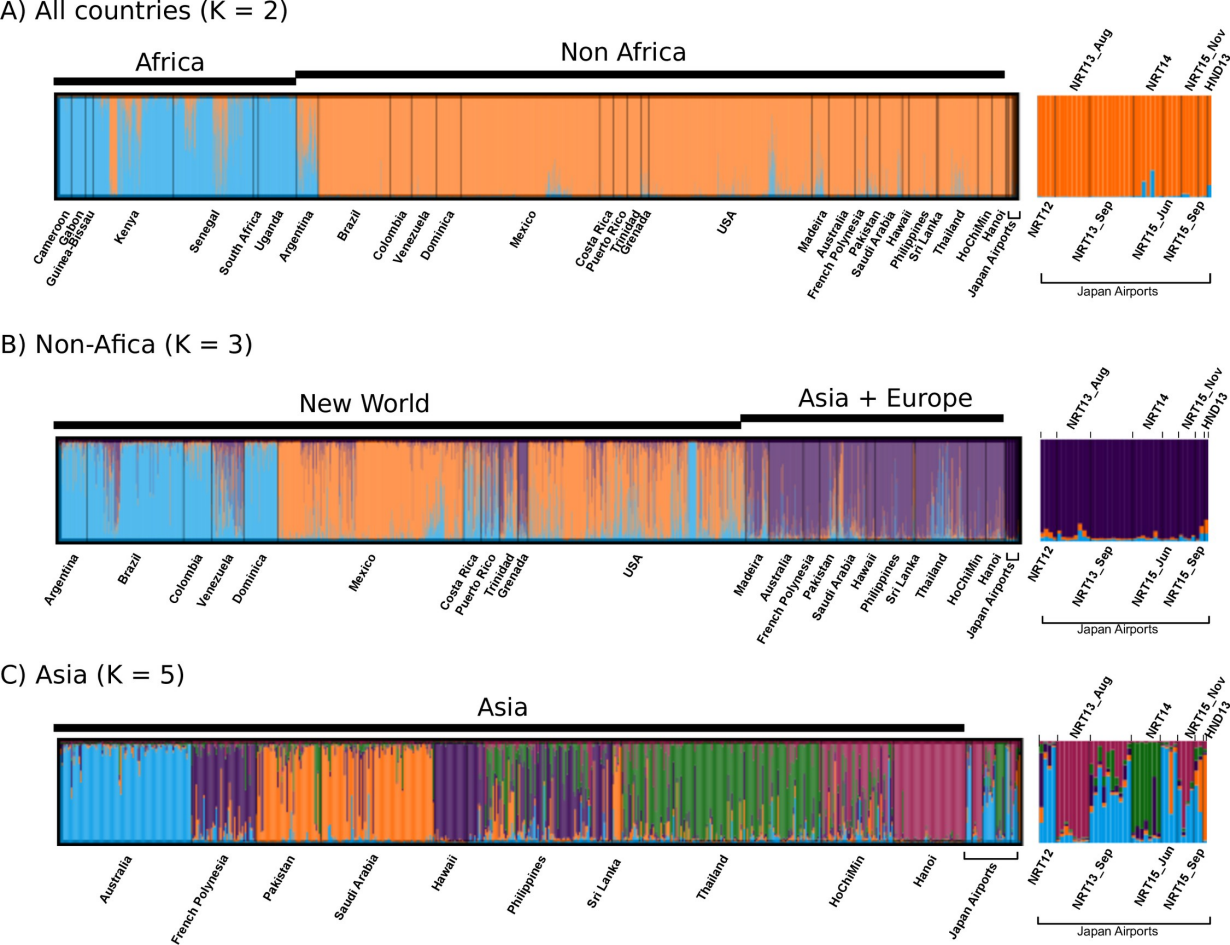
Bayesian clustering by STRUCTURE. The result of multiple STRUCURE runs were averaged by CLUMPP. Only results for the best K-values in Evanno’s method are shown. Magnified views for the airport samples are shown at the right end of each figure. (A) Clusters of all *Ae*. *aegypti* genotypes + airport samples in Japan at the best k-value = 2. (B) Clusters of out-of-Africa genotypes + airport samples in Japan at the best k-value = 3. There were two distinct clustering results. (C) Clusters of Asia/Pacific genotypes + airport samples in Japan at the best k-value = 5.

**Fig 3:**
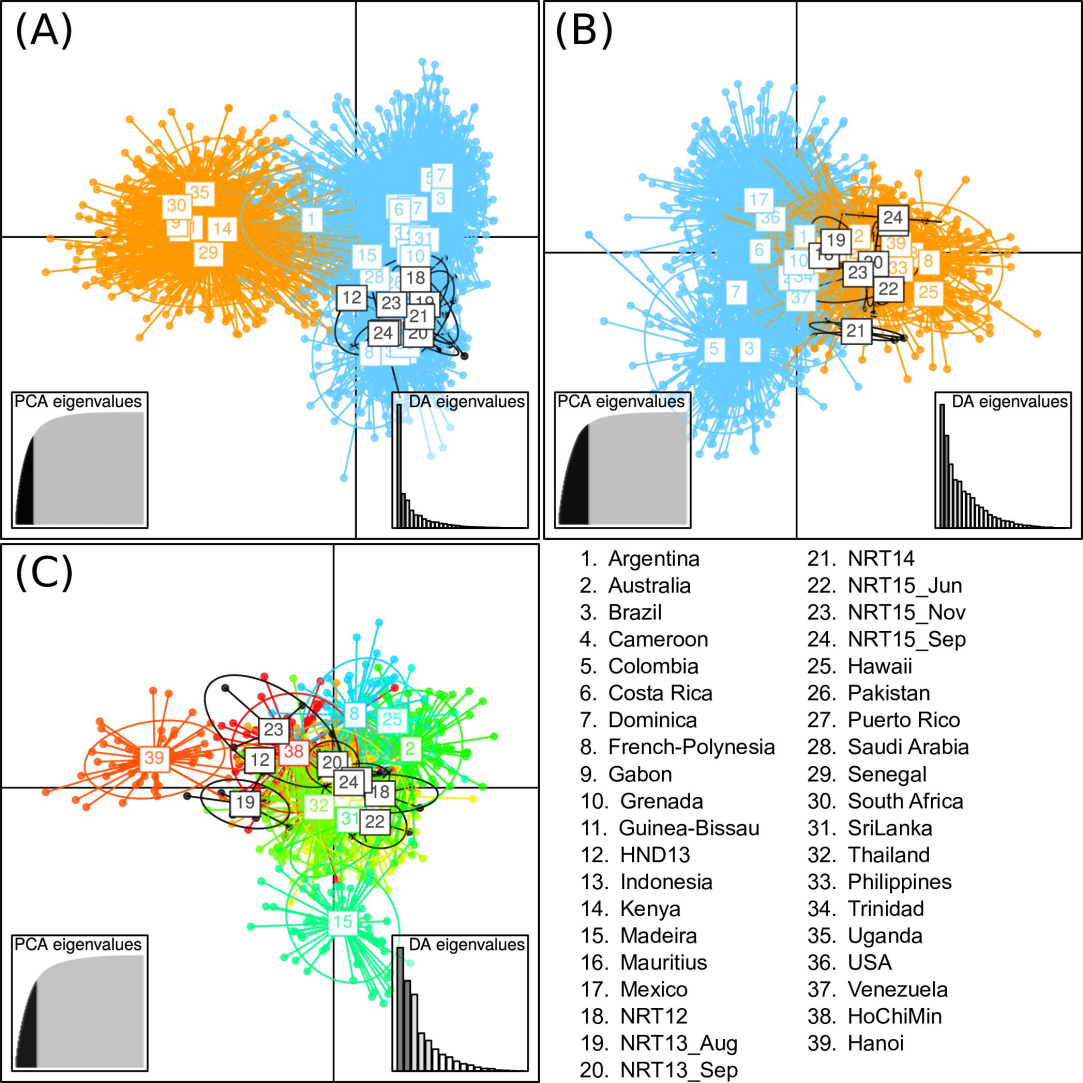
DAPC by adegenet. The results of the DAPC are shown. The X- and Y- axes indicate the 1^st^ and 2^nd^ principal component of DAPC, respectively. The right and left insets show scree plots of PCA and DA eigenvalues, respectively. The labels and individual points for airport samples in Japan are drawn in black. First, we conducted clustering using whole genotype data altogether with airport samples in Japan (A). Next, out-of-African genotypes together with airport samples in Japan were clustered. (B). Finally, Asia/Pacific genotypes along with airport samples in Japan were clustered (C).

The 40 *Ae*. *aegypti* collected at two international airports serving the Greater Tokyo Area during 2012–2015 were genotyped for the 12 microsatellite loci. As confirmed in preceding study [2,7], STRUCTURE separated the whole individual genotypes (samples in this study + references) into Africa and out-of-Africa genetic clusters at the best k-value = 2, with some level of admixture in Kenya and Argentina (Fig 2A). All samples collected at the airports belonged to the out-of-Africa cluster. The genotypes from out-of-Africa countries plus the airport samples were further separated into two New-World clusters and one Asia/Pacific cluster at the best k-value = 3 (Fig 2B) as already seen in the previous study [7]. The genotypes of the airport samples showed preference to the Asia/Pacific cluster. The Asia/Pacific group plus the airport samples were separated at the best k-value = 5 (Fig 2C). The results revealed a weak population structure according to geographic locations/countries. Although the airport mosquitoes captured in same incident had similar posterior probabilities for each genetic cluster, pattern for mosquitoes captured in different incident showed affinities to several different clusters. In particular, NRT12 and NTR15_Jun, NRT13, NRT14, and the sole HND13 individual were clustered into Australia, Vietnam-Hanoi, Thailand and Middle-East/Sri Lanka clusters, respectively, with relatively high posterior probabilities (Fig 2C). The DAPC also supported a membership of the airport samples within the out-of-Africa group (Fig 3A). Clustering genotypes excluding Africa data marginally separated New-World and Asia/Pacific genotypes with substantial overlap. All airport samples were contained within the range of Asia/Pacific cluster (Fig 3B) but were not completely distinct from the New-World cluster. No additional fine clustering was obtained from the DAPC within Asia/Pacific group (Fig 3C).

The results of STRUCTURE and DAPC were cross-validated by assigning the airport samples to *a priori* defined genetic groups using GeneClass2. All airport samples, with the exception of one in NTR14, were assigned to the out-of-Africa group in the “Africa or out-of-Africa” selection panel. When using the “New-World or Asia/Pacific” selection panel, most individual genotypes in NRT samples showed an Asia/Pacific origin. The sole individual from HND13, on the other hand, was assigned to the New-World group (S1 Fig).

### Mitochondrial lineage

**Fig 4:**
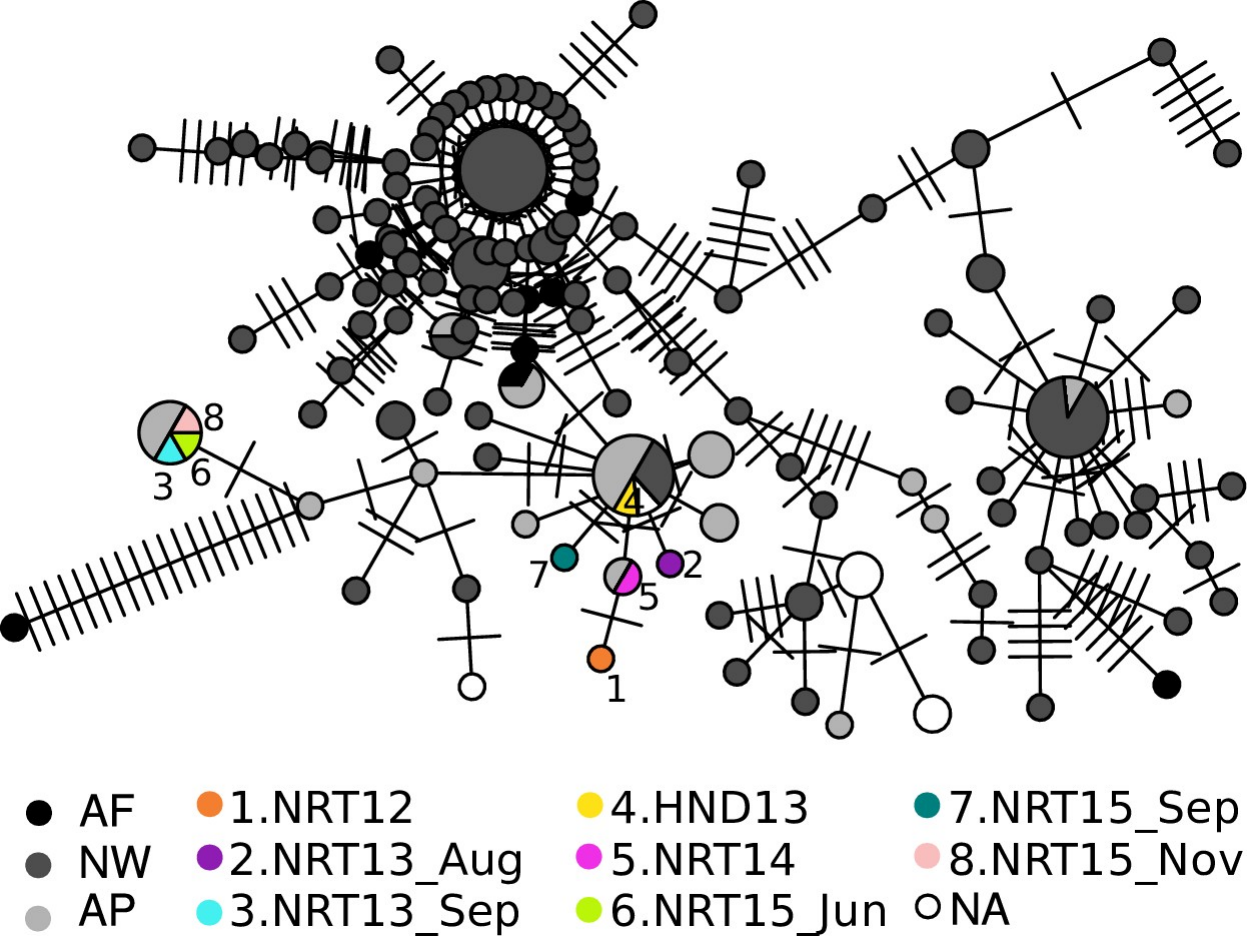
Haplotype network for the *Cox1* gene. Each node indicates a distinct *Cox1* haplotype. The number of ticks on each edge indicate the number of mutations. AF, Africa, NW, New-world, AP, Asia/Pacific, NA, Information not available.

The mitochondria *Cox1* gene was sequenced in the 40 *Ae*. *aegypti* individuals captured at airports. Individuals captured during the same incident had identical haplotype. Fig 4 depicts the haplotype network for the *Cox1* haplotypes of each incident, in addition to the other entries retrieved from the NCBI Nucleotide collection database. NTR13_Sep, NRT15_Jun and NRT15_Nov mosquitoes shared the same haplotype which was also identical to haplotypes reported from Asia/Pacific region (Fig 4). Although other airport samples each had unique haplotype among all airport samples, the haplotypes in HND13 and NRT14 were identical to haplotypes reported previously for the Asia/Pacific region and both New-World and Asia/Pacific region, respectively.

## Discussion

### Origin of the *Ae*. *aegypti* mosquitoes captured at the Narita and Haneda international airports

We analyzed *Ae*. *aegypti* mosquitoes captured in two international airports serving the Greater Tokyo Area. Both Bayesian (STRUCTURE) and multivariate (DAPC) clustering methods supported the conclusion that all individuals belonged to the Asia/Pacific genetic group. Although GeneClass2 assigned most individuals to the Asia/Pacific group using a hierarchical clustering approach, one individual was clustered into the Africa cluster in the “Africa or out-of-Africa” selection panel and one sample from NRT15_Nov and the sole HND13 individual were clustered into the New-World cluster using the “New-World or Asia/Pacific” selection panel with high probability (>80%) (S1 Fig). During 2012 to 2015, more than half of the total passenger planes arriving at Narita Airport originated from the Asia/Pacific region, while direct flights originating from Africa or South America (most likely source in the New-World) accounted for less than 0.3% of the total flights [31]. Considering the relatively high misassignment rate in the GeneClass2 test (see Materials and Methods) and the high traffic volume from the Asia/Pacific regions into Japan, at the moment, we assume that the origins of all airport samples are somewhere in Asia/Pacific region. Although STRUCTURE analysis classified some individuals into more specific clusters (Fig 2C) with relatively high posterior probabilities, these results should remain speculative because the number of Asian and Pacific countries represented in the reference panel is limited. To obtain increased confidence and resolution to assign individuals into narrower local populations (i.e., country level), further expansion of reference panel to include additional worldwide populations and the utilization of richer genetic information, such as genome-wide SNPs [32–34] will be required.

### Are *Ae*. *aegypti* mosquitoes reproducing stably in airports in Japan?

The mosquitoes collected in the same incident had identical mitochondrial haplotypes. Conversely, mosquitoes collected in different incidents had different mitochondrial haplotypes indicating that there were multiple different maternal lineages for *Ae*. *aegypti* mosquitoes collected in airports during 2012–2015. Furthermore, the STRUCTURE analysis did not assign all airport individuals into a single cluster within the Asia/Pacific group. Considering the fact that the discoveries were occasional and intensive follow-up following after discovery did not find additional *Ae*. *aegypti*, there is little evidence to support the establishment of stable *Ae*. *aegypti* populations in airports.

While most regions in Japan are not suitable for *Ae*. *aegypti* inhabitation, this species once established an overwintering population in temperate zone in Japan within a limited period after World War II (1944–1952) [35]. Overwintering of *Ae*. *aegypti* was also suspected in Washington, DC during 2011–2014, where the mosquitoes may have been utilizing subterranean habitats [36]. In 2014, a local infection of dengue occurred in Tokyo [37] for the first time in 70 years, though the vector mosquito was different species of mosquito, *Ae*. *albopictus*. Nevertheless, the continuous introduction of both vectors and pathogens poses an undesirable risk that would change the epidemiological situation in this country. Thus, further intensive surveillance and preventive measures for exotic mosquitoes in airports are desired.

## Supporting information

S1 File Primer and dye combination used for microsatellite amplification. (XLSX)

S2 File Microsatellite genotypes of each mosquito collected in airports. (TSV)

S1 Fig Results of the GeneClass2 analysis. (PDF)
